# BEHAVIORAL HEALTH PREDICTORS OF PREMATURE MORTALITY IN A BLACK AMERICAN LONGITUDINAL COHORT

**DOI:** 10.1093/geroni/igad104.2344

**Published:** 2023-12-21

**Authors:** Kerry Green, Brittany Bugbee, Elaine Doherty

**Affiliations:** University of Maryland at College Park, College Park, Maryland, United States; University of Maryland School of Public Health, College Park, Maryland, United States; University of Maryland, College Park, Maryland, United States

## Abstract

Life expectancy among Americans is now the lowest it has been in 15 years. While startling, more alarming are the persistent racial disparities in mortality. Black Americans continue to die at significantly younger ages with Black men living, on average, 7.0 fewer years and Black women living 4.4 fewer years than their White counterparts. To inform intervention strategies and resource allocation, we need a complete understanding of the role that malleable factors, such as substance use and depression early in the life course, play in premature mortality for aging Black Americans. This study illuminates behavioral health factors that can be targeted to eliminate mortality disparities among a longitudinal cohort followed from childhood into their 60s. Utilizing data from the Woodlawn Study, a well-defined neighborhood cohort study of 1,242 Black Americans that began in 1966 (age 6), we examined the predictive value of family history of substance use, adolescent substance use and depression, and young adult substance use and depression. Through 2021 (modal age 61), based on National Death Index reports, 21.6% of the original cohort (n=268) has passed away. The poster will present cause and timing of death by gender and adjusted logistic regression findings showing that depression symptoms as early as adolescence (age 16) predict midlife mortality occurring between ages 32 and 60 (p=.003) and that those diagnosed with depression or a substance use disorder in young adulthood are 61% (p=.025) and 85% (p=.001) more likely to die by age 60. Policy and prevention implications will be presented.

